# Advances and challenges in studying effects of EDCs on tissue-resident macrophages in inflammation

**DOI:** 10.1210/jendso/bvag044

**Published:** 2026-02-27

**Authors:** Margaret R Bell, Katherine A Walker, Gia M Valdez, Carissa E Dressel

**Affiliations:** Department of Biological Sciences, DePaul University, Chicago, IL 60614, USA; Departments of Health Sciences and Neuroscience, DePaul University, Chicago, IL 60614, USA; Department of Chemistry, Purdue University, West Lafayette, IN 47907, USA; Department of Biological Sciences, DePaul University, Chicago, IL 60614, USA; Department of Environmental Medicine & Public Health Sciences, University of Rochester Medical Center, Rochester, NY 14642, USA

**Keywords:** EDCs, PCBs, macrophages, inflammation, adipose, microglia

Polychlorinated biphenyls (PCBs) have long been studied as endocrine-disrupting chemicals (EDCs) and are associated with a range of disorders, including metabolic syndrome, reproductive dysfunction, and altered neurodevelopment [1]. The 200+ individual congeners differ in their mechanisms of action, as coplanar “dioxin-like” congeners bind aryl hydrocarbon receptor (AhR) while non-coplanar compounds interact with a range of nuclear receptors, cellular proteins, and ion channels, and promote reactive oxygen species production. Because humans are exposed to mixtures of compounds, and a single congener can have several mechanisms, integration of endocrine disruption with other forms of toxicity is critical. Many PCB-associated health outcomes are associated with inflammation, which may be an intersection of multiple modes of action.

Macrophages are important innate immune cells that are responsive to a range of environmental contaminants, including persistent organic pollutants, heavy metals, and airborne particulate matter. In their recent research published in the *Journal of the Endocrine Society*, Behan-Bush et al study the effects of a widespread industrial PCB mixture called Arocolor 1254 on macrophages derived *in vitro* from human peripheral blood mononuclear cells [2]. They demonstrate that exposure to A1254 shifts macrophages into pro-inflammatory M1, and away from M2a and M2c anti-inflammatory, phenotypes. A strength of the work is the attention to the process of polarization by exposing cells to PCBs prior to and during addition of differentiating factors, as PCBs are likely already present in the tissues while they are developing or responding to inflammatory challenge. This complements the group's recent work showing that PCB mixtures can also shift previously differentiated cells toward inflammatory and away from anti-inflammatory states. Given the dynamic plasticity of macrophages, consideration of temporal and phenotype-specific processes is essential. Indeed, the authors illustrate that while effects of A1254 on surface receptor expression and cytokine release in M2a and M2c cells are dependent on AhR action, effects on glucose metabolism in M2c differentiated cells are not. One plausible explanation for this is the engagement of glucocorticoid receptor activity, as dexamethasone is used to drive the M2c phenotype. That the A1254 mixture appears to affect glucose metabolism via more than one molecular process affirms the challenges of studying PCBs and mixtures.

In addition to cytokine release, a key aspect of macrophage function is phagocytosis of pathogens, damaged cells, debris, or foreign particles for removal. Although phagocytosis was not directly measured in this study, several surface markers important in phagocytosis, such as CD14 and CD16, were altered by PCB exposure, suggesting a potential change in capacity. Defective macrophage phagocytosis is associated with cardiovascular and metabolic, pulmonary, and neurodevelopment and neurodegenerative disorders. Yet, how environmental contaminants reprogram phagocytic capacity remains unresolved and is an area for continued analysis.

Another strength of Behan-Bush's approach is the use of primary human monocyte-derived cells, as AhR signaling shows some species-specific processes. In addition, macrophage cells lines, an alternative model, do not always replicate primary responses to inflammatory and lipid signaling. Of course, using a diversity of models to understand the effects of environmental contaminants on health is essential, as each has strengths and limitations. In this publication, an *in vitro* model allows for testing A1254 mechanisms of action directly on macrophages, by preventing effects of exposure with AhR antagonists and demonstrating dose-dependent recapitulation of effects by coplanar PCB 126. At the same time, the study of PCBs on macrophage cell function *in vivo* allows for analysis to incorporate effects of PCBs in native tissues and interacting systems [3]. An additional strength of the model used by Behan-Bush is that individual patient cells were tested in parallel across treatment in a paired design. The authors provide details of patient demographics, which included male and female individuals of different ages. This may explain the variability in degree of response to treatment across samples and highlights the need for appropriately powered sex- and developmental period–specific analysis.

While Behan-Bush et al tested monocyte-derived macrophages as targets of PCBs, the contextual motivation behind these experiments concerns adipose-resident macrophages. Adipose tissue is recognized as a site of lipophilic toxicant accumulation and a target of obesogens [4]. Lipid droplets and the lipid species contained within can directly influence macrophage activity, including phagocytic efficiency. However, studies often focus on effects on adipocytes or systemic inflammation, rather than how EDCs directly reshape tissue-resident macrophage polarization, signaling, and downstream consequences [5]. Because adipose-immune signaling is a critical determinant of metabolic function in type 2 diabetes, cardiovascular disease, and nonalcoholic fatty liver disease, understanding effects of contaminant exposure on local macrophages is key. As such, models that incorporate tailored differentiation protocols to include pro-inflammatory hypertrophic adipose-associated cytokines and lipids, co-culture with adipocytes or mesenchymal stem cells, or derivation of cells from specific patient adipose depots or disease states are uniquely powered to detect real-world effects of environmental contaminants on disease risk.

As Behan-Bush et al note, this published work has implications for other lipid-rich tissues like the brain. Like adipose tissue, the brain can serve as a reservoir for PCBs and is highly responsive to endocrine and immune signaling. The brain's resident macrophages, microglia, are also in constant conversation with neighboring cells and shift into a range of phenotypic states over development and in response to environmental challenges. While some studies do not detect major effects of PCB exposure on microglial function, designs that interrogate the effects of PCBs on later microglial responses to secondary challenges detect age- and sex-specific effects (eg, Walker et al, 2024 [6]). Recent findings regarding lipid signaling and crosstalk between brain and adipose tissues reveal further parallels and interactions. For example, lipid dysfunction in both adipose and brain macrophages are associated with disease [7] and microglial signaling can affect adipose tissue function in models of obesity [8]. Altogether, studies of environmental contaminant effects on tissue-resident macrophages that, like Behan-Bush et al, address cell plasticity and development provide insights relevant to a range of chronic disease processes.

## Abbreviations

AhR: aryl hydrocarbon receptor
EDC: endocrine-disrupting chemical
PCB: polychlorinated biphenyl
